# Regulating Cross-Linking Structure and Dispersion of Core-Shell-Rubber Particles in Polyurethane Composite to Achieve Excellent Mechanical Properties for Structural Adhesive Application

**DOI:** 10.3390/polym16233263

**Published:** 2024-11-23

**Authors:** Zijin Jiang, Lingtong Li, Luoping Fu, Yingte Xu, Lin Zhang, Hong Wu, Shaoyun Guo

**Affiliations:** 1 The State Key Laboratory of Polymer Materials Engineering, Polymer Research Institute of Sichuan University, Chengdu 610065, China; jiangzijin@dongfang.com (Z.J.); lingtongli1994@163.com (L.L.); nic7702@scu.edu.cn (S.G.); 2 Sichuan Dongshu New Materials Co., Ltd., Deyang 618000, China; fuluoping@outlook.com (L.F.); xu1998yt@outlook.com (Y.X.); dy_zlin@163.com (L.Z.)

**Keywords:** polyurethane, cross-linking, mechanical property, structural adhesive

## Abstract

Structural adhesives are bonding materials that can quickly join structures with components and repair cracks. However, thermosetting polyurethane structural adhesives suffer from disadvantages such as insufficient toughness, poor aging resistance, and long curing time, which greatly limit their practical application. Herein, a polyurethane (PU) composite with excellent mechanical properties was prepared successfully via regulating the cross-linking structure and the dispersion of core-shell-rubber (CSR) particles. Various polyols were selected to improve the cross-linking density of the PU and to enhance the intermolecular forces, which can achieve the high strength and stability of the polyurethane composites. Solvent displacement was used to improve the dispersion of CSR in PU. The cured composite has ultra-high toughness and impact resistance due to the well-dispersed CSR particles. The impact strength was increased from 52.0 to 90.4 kJ/m^2^, and the elongation at break was increased from 6.1% to 14.9%. Due to the addition of catalyst T120, this composite can be cured quickly at room temperature, reaching high strength after 30 min. In addition, these composites can resist extreme environments, such as high and low temperature changes, UV aging, high humidity and heat environment, and salt spray aging, which has potential and value for practical application. The prepared PU structural adhesive can meet the requirements of structural bonding transit and improve the production efficiency. This work proposed a novel strategy to prepare polyurethane composites with excellent mechanical properties for structural adhesive application.

## 1. Introduction

Strengthening structures are being used widely with the increase in outdoor facility constructing and remodeling [1,2]. Mechanical fastening [3], welding [4], and structural adhesive bonding [5] are primary techniques for joining dissimilar materials. Traditional joining of materials relies mainly on mechanical fastening. However, it is prone to stress concentration, heat effects, tiredness, and corrosion. Technologies such as laser [6], ultrasonic [7], and friction stir welding [8] are promising today. In polymers, however, the application of these materials is limited to thermoplastics [9,10]. In contrast, structural adhesive has advantages, including good static-dynamic loading, high damage tolerance, evenly distributed stress, low cost, and quality, which can be used for the repairing and bonding of different materials [11,12,13,14]. Pitta et al. [15] compared adhesive-bonded lap joints between metal-metal and metal-composite configurations with metal-metal riveted lap joints, demonstrating that the adhesive joints exhibited an average strength three times greater than that of the riveted joints. Therefore, effective strengthening of structural materials through adhesive bonding is actually one of the best options [16,17,18].

Various types of adhesives can be utilized in structural applications, mainly including epoxy [16,19,20], acrylate [21,22], and polyurethane (PU) [23,24]. Currently, various types of these materials have different problems that limit their practical application as structural adhesive bonding materials [25]. In particular, epoxy structural adhesives are usually brittle and have poor aging resistance and long curing times. Acrylate structural adhesives have high shrinkage, high brittleness, and even toxicity. PU-based structural adhesives have the disadvantages of insufficient toughness, long curing times, and are not suitable for fast curing. However, the properties of PU materials can be adjusted by adjusting the type of reaction in order to regulate the ratio of soft to hard segments in the polymer chains [13,26]. The chemical properties of PU adhesives have been extensively studied, demonstrating significant multifunctionality and raising their use [27]. This material has been shown to be capable of use in structural applications in recent studies [1]. It has been a popular research focus on how to effectively regulate the cross-linking structure in PU structural adhesives to achieve excellent mechanical stability and weatherability.

PU adhesive has become one of the most diverse and productive structural adhesives due to its adjustability of properties, high adhesive strength, and wide adhesive range in recent years [5,14]. Besides effective regulation of the cross-linking structure in PU, the introduction of particles could also achieve excellent properties. Mehmet Emin Çetin [28] investigated the low-velocity impact behavior of PU adhesive sandwich panels, and the results indicated that the addition of MWCNT greatly enhances the impact resistance. Saeed Habibpour et al. [29] investigated the PU-filler with different nanoparticle loadings and found the PU-graphene nanoribbon (GNR) nanocomposites improve load-bearing properties. Currently, a large number of investigations report the multifunctionality of PU composites for different scenarios [30]. However, efficiently regulating the cross-linking structure in PU materials while accurately controlling the good dispersion of particles is still a challenge.

In this work, we successfully prepared a PU composite with good mechanical properties by effectively regulating the cross-linking structure of the PU material and the dispersion of the core-shell-rubber (CSR) particles. High structural strength and toughness can be achieved by curing at room temperature for 30 min. Different kinds of polyols can increase the cross-linking curing density, enhance the intermolecular forces, and achieve high strength and stability. Well-dispersed CSR particles give the composite ultra-high toughness and impact resistance after curing. The prepared PU composites show good mechanical properties and stability after various weathering tests. When this composite is fully cured as a structural adhesive, it has a smooth and uniform surface, good densification and basically no shrinkage, which can meet the requirements of structural bonding transit and improve production. This work presents a novel strategy to prepare PU composites with excellent mechanical properties and weatherability for structural adhesive application.

## 2. Materials and Methods

### 2.1. Materials

Polymerized isocyanate diphenylmethane diisocyanate (MDI, PM200) was purchased from Wanhua Chemical Polyurethane Co., Ltd. (Yantai, China). Polyether polyols DDL1000D and MN500 were purchased from Zibo Dexin Federal Chemical Co., Ltd. (Zibo, China). Cashew nutshell oil polyol NX9008, hyperbranched polyol NJ403, and heat-resistant polyol HP2210 were purchased from Cardolite Chemical Co., Ltd. (Zhuhai, China), Jurong Ningwu New Material Co., Ltd. (Jurong, China), and Shanghai Yinman Chemical Co., Ltd. (Shanghai, China), respectively. Core-shell-rubber (CSR) particle FD3110 consisting of a polymethylmethacrylate (PMMA) shell and a polybutadiene rubber core was purchased from Nortek New Material Co., LTD. (Shanghai, China). Chain extender 4, 4-methylene-bis(2-chloroaniline) (MOCA) was purchased from Xiangyuan New Material Co., LTD. (Suzhou, China). The curing of PU can be accelerated by appropriate amounts of catalyst T120, which were purchased from Evonik Industries AG. (Beijing, China). Reagent BYK-X (defoamer BYK-066, leveling agent BYK-333, and dispersant BYK-9076) was purchased from BYK Chemical Co., Ltd. (Tonglin, China). Carbon black BR301 was purchased from Tianjin Yiborui Chemical Co., Ltd. (Tianjin, China). Butanone (C6H12O, AR, 99%) was provided by Chengdu Colon reagent factory. All reagents were used as received without further purification.

### 2.2. Preparation of PU Composites

**Figure 1:**
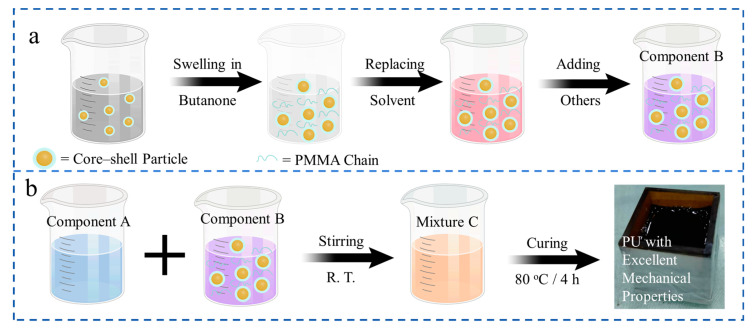
Schematic illustration of: (**a**) component B and (**b**) PU composite fabrication process.

Initially, the reactor was heated to 80 °C and dried for 3 h, after which the required amount of polymerized isocyanate MDI (PM200) was introduced. It was stirred to obtain component A in N_2_. Secondly, appropriate amounts of CSR particles (FD3110) were dissolved and dispersed in butanone solvent with different times in another dry reactor. Then, appropriate amounts of polyether polyols (DDL1000) were added to displace the CSR particles dispersed in butanone. Later, the butanone was removed by distillation under reduced pressure to obtain homogeneously dispersed CSR particles with a polyether polyol component. Then, appropriate amounts of polyols (DMN40, NX9008, NJ403, and HP2210) were added, respectively. Stirring was applied at 80 °C for 30 min, followed by cooling to 30 °C. Then, chain extenders (MOCA), catalysts (T120), reagent (BYK-X), and carbon black (BR301) were added and stirred well to obtain component B. The process above is shown graphically in Figure 1a. The swelling of PMMA shell in butanone resulted in an increase in the size of the CSR particles and exposure of more hydroxyl groups, which regulated the dispersion in component B. The particle size distribution of the CSR particles before and after swelling was characterized by a laser particle size shape analyzer, as shown in Figure S1. Photographs of the dispersion of the CSR particles in component B before and after swelling are also shown in Figure S1.

Components A and B were mixed to produce mixture C, which was then prepared for processing. The ratio of components A and B was controlled to regulate the network structure of the PU composites and the distribution of the CSR particles. The compositions of component A and B are shown in Table 1, and the composition of PU composites is shown in Table 2. In this work, the composition of component B is constant. The amounts of CSR particles (FD3110) and polyols are varied simultaneously in the preparation of different PU composites. We regulated the molar fraction of –OH and –NCO groups to be 1:1.05 to ensure complete reaction in S0 and S3 samples. The minor amount of NCO is to meet the reaction with water in the air. The S0 sample was prepared as a comparison by removing the CSR particles based on the S3 sample. After mixing and stirring, the PU composites can be quickly surface dried at room temperature and then placed at 80 °C for 4 h to be fully cured, obtaining PU composites with good mechanical properties, as shown in Figure 1b. The components participating in the reaction required vacuum dehydration prior to the reaction.

### 2.3. Characterization

The particle size distribution of the CSR particles was characterized by a laser particle size and shape analyzer (SYNC, Microtrac, Largo, FL, USA). The structure of the CSR particle was characterized by the transmission electron microscope (TEM, JEM-1400 Flash, Japan Electronics Corporation, Tokyo, Japan). A scanning electron microscope (SEM, Nova Nano 450, Hillsboro, OR, USA) was used to investigate the micro cross-sectional surface morphologies of the PU composites. An infrared spectrometer (IS10, Thermo Nicolet, Waltham, MA, USA) was used to obtain the attenuated total reflection–Fourier transform infrared spectra (ATR–FTIR) of PU composites at room temperature. The number of scans, FTIR resolution, and scanning range were 32, 4 cm^−1^, and 4000–400 cm^−1^, respectively. The mechanical properties of the samples were tested by a material testing machine (MST, INSTRON 5966, Canton, MA, USA). Samples were stretched in the length direction with the stretching rate of 1 mm/min, and the average was calculated from 5 samples with the same size (4 mm × 10 cm × 60 cm). The impact strength was tested by a pendulum impact tester (MST, ZBC8501-B, Canton, MA, USA). For dynamic mechanical analysis (DMA), PU samples were prepared by casting with the same size (20 mm × 10 mm × 4 mm). DMA data were investigated by a Q800 instrument (TA Instruments, Newcastle, DE, USA) at 1 Hz and 0.01% strain. The test temperature was from −70 to 200 °C (or until samples yielded) at a heating rate of 3 °C/min. High- and low-temperature change was tested by a high- and low-temperature chamber (BPHJS-120C, Shanghai Yiheng Technology Co., Ltd., Shanghai, China) by raising the temperature to 80 °C for 5 h (90% humidity), then turning down to −40 °C for 5 h. The UV aging was tested in a UV aging chamber (QUV/Spray, H. J. UNKEL FOSHAN Ltd., Foshan, China). Samples were exposed for 5 h with selective UV intensity (1.55 W/m^2^), wavelength (340 nm), and temperature (8 °C). The samples were then further irradiated at 20 °C and sprayed with water for 1 h. The 85 humidity and 85 temperature tests were tested by a high- and low-temperature chamber (GDW JS-800, Shanghai Suying Test Instrument Co., Ltd., Shanghai, China). Salt spray aging test was tested by (Q-FOG/SSP600, H. J. UNKEL FOSHAN Ltd., Foshan, China). In order to verify the practical application potential of this PU composite, we poured the prepared PU sample into a self-made metal frame. The structural adhesive block was carefully separated from the metal frame and then tested for dimensional differences between the frame and the structural adhesive.

## 3. Results and Discussion

### 3.1. Fast Curing Performance and Characterization of PU Composites

**Figure 2:**
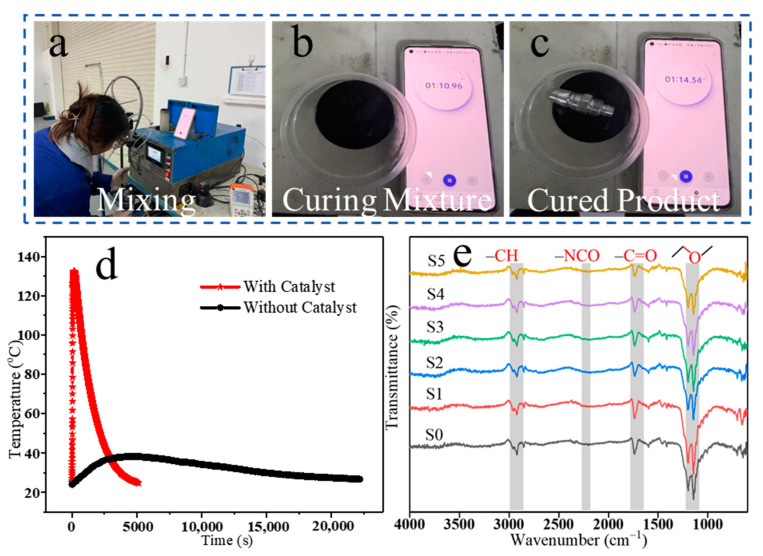
(**a**–**c**) Digital photographs of the S3 sample mixing process; (**d**) gel exothermic curve of the S3 sample after mixing; (**e**) FT-IR spectrum of S0–S5 samples.

The fast curing of structural adhesives is important for practical expansion of the applications. In this work, we can achieve both fast curing and good mechanical properties of PU structural adhesives. Due to the addition of catalyst T120, the reactivity of PU mixtures increases, and the reaction rate is accelerated comparing to the reaction without T120. Figure 2a–c shows the fast preparation process of a PU sample. We can notice that the mixture achieves a fast curing of the sample surface after only 75 s, and the surface cures without sticking to other materials. Figure 2d shows the gel exothermic curve of the S3 composite with and without T120 after mixing. The exothermic peak of the composite with catalyst T120 (red curve) is about 110 s, while the exothermic peak of the composite without catalyst (black curve) is flat and the reaction is slow. This further illustrates the fast curing process of the mixture. A fast curing process of the structural adhesive is important, as are the mechanical properties of the material after curing. More than 70% of the structural strength can be achieved after 30 min at room temperature, as shown in Figure S3. The prepared PU structural adhesive can meet the requirements of structural bonding transit and improve the production efficiency. As shown in Figure 2e, we characterized the PU composites by FT-IR spectrum. The signal observed in the range of 2825–3000 cm^−1^ corresponded to the antisymmetric and symmetric vibrations of –CH_n_ groups, where n can be 1, 2, or 3 [31]. The absorption peak observed in the range of 1750–1660 cm^−1^ can be ascribed to the vibration of the carbonyl group. The bending vibration peak of the –NH group was observed at 1530 cm^−1^ [32]. The wide signal around 1110 cm^−1^ can be attributed to the vibration of the C–O–C group. Series of peaks found in the 950–790 cm^−1^ range correspond to the C–H vibrations of the benzene ring. The stretching vibration peak of N=C=O at 2254 cm^−1^ disappeared [33]. The results above confirmed the successful preparation of PU composites.

### 3.2. Mechanical Properties of PU Composites

**Figure 3:**
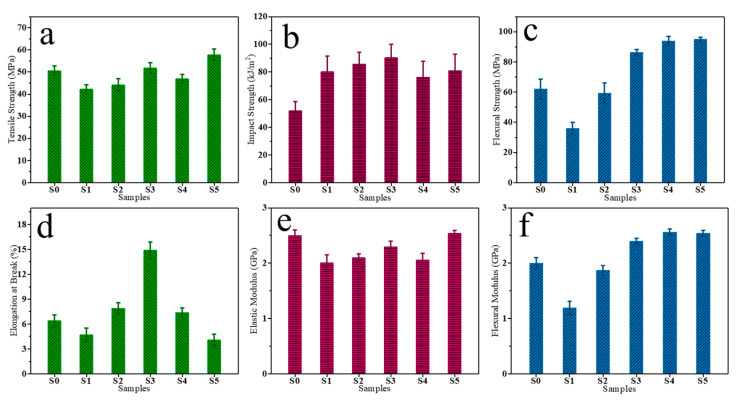
(**a**) Tensile Strength, (**b**) impact strength, (**c**) flexural strength, (**d**) elongation at break, (**e**) elasticity modulus, and (**f**) flexural modulus of different PU composites at 25 °C.

To evaluate the mechanical performance of PU composites, the mechanical properties of the pouring samples were analyzed at room temperature. The test results are presented in Figure 3a–f, with detailed data provided in Table S1. The various mechanical properties of the PU samples showed different trends of increase and decrease with increasing B-component content. In comparison, the tensile strength, impact strength, and elongation at break of S3 samples were the highest among all the samples, which were 51.8 MPa, 90.4 kJ/m^2^, and 14.9%, respectively. The cross-linking structure of PU provides a large number of reinforcing networks and well-dispersed CSR particles, which greatly improve the tensile and flexural strength of the polyurethane material. The flexural strength, flexural modulus, and elastic modulus of S3 samples were also in the upper level among all samples; respectively, 86.3 MPa, 2.4 GPa, and 2.3 GPa. Compared with the S0 samples without CSR particles, the impact strength and flexural strength of S3 samples were greatly increased. The well-dispersed CSR particles could dissipate energy and improve impact fracture toughness. This strong and tough S3 sample material greatly expands the use of PU composite as a structural adhesive.

**Figure 4:**
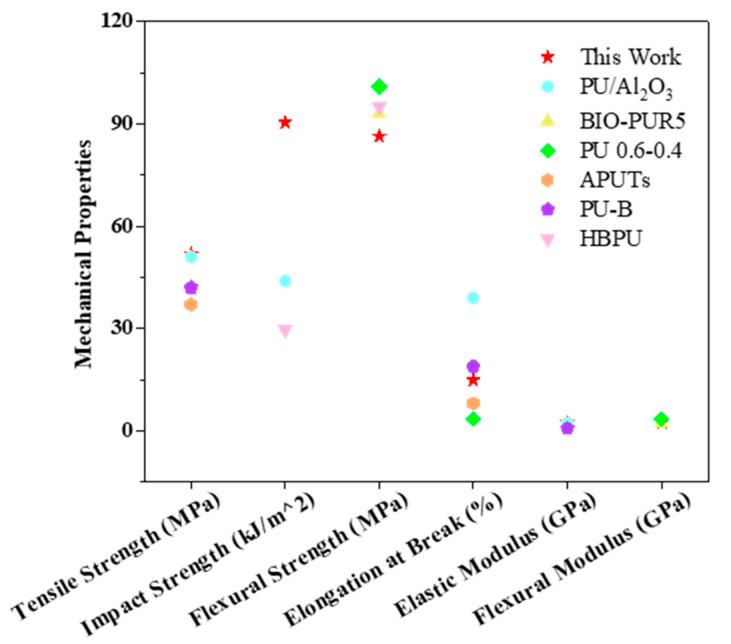
Comparison of the mechanical performance of prepared PU composites in this work with other PU in the literature.

As can be observed from Figure 4, the PU composites prepared in this work exhibited comparable or higher tensile strength, impact strength, flexural strength, and elongation at break than most thermosetting PU composites reported in the literature [34,35,36,37,38,39]. The attractive features of the developed PU composites, such as good and easily tunable mechanical performance, including a broad range of flexural strength (36–95 MPa), impact strength (75–90 MPa), and tensile strength (42–57 MPa), together with a fast curing process and good weathering resistance, could cement these PU composites as a promising class of high-performance PU materials.

**Figure 5:**
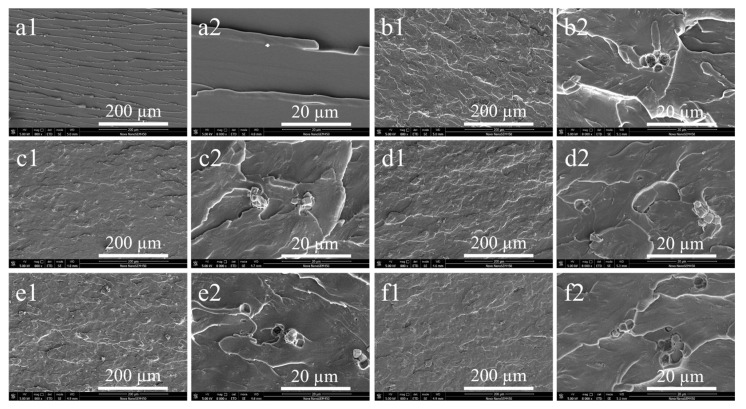
SEM of notched impact cross-sectional surfaces of different PU samples with different magnifications: (**a1**,**a2**) S0, (**b1**,**b2**) S1, (**c1**,**c2**) S2, (**d1**,**d2**) S3, (**e1**,**e2**) S4, and (**f1**,**f2**) S5.

To further investigate the toughening effect of the CSR particles on the PU composites, we used scanning electron microscopy analysis of the impact cross-sectional surfaces for different samples, as shown in Figure 5. With the increasing component B, more and more CSR particles appeared in these sections. These CSR particles have good dispersion and uniform distribution, and it was difficult to observe a significant agglomeration in all the samples. In the S3, S4, and S5 samples, we can observe a clear pull-out of toughened particles. In the S3 sample, there is a fuzzy interfacial connection between the CSR particles and the PU matrix, which proves that the toughened particles have good compatibility with the PU matrix. In addition, we observed the crack structure appeared in the S0 section without CSR particles. This may be attributed to the stress needed for brittle fracture following the incorporation of different polyols. These observations confirm the good toughening effect of the CSR particles.

### 3.3. Thermal Stability of PU Composites

**Figure 6:**
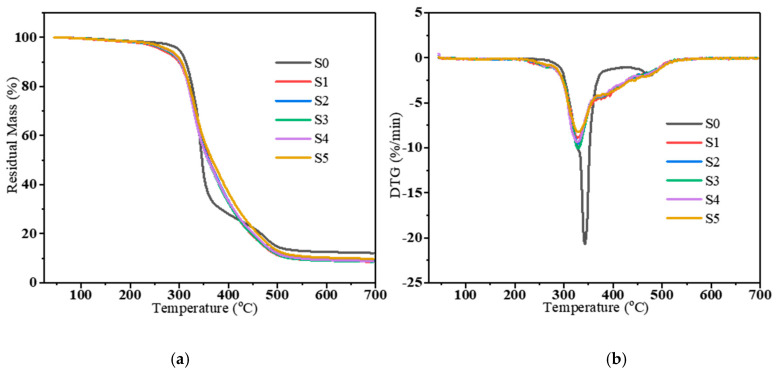
TGA (**a**) and DTG (**b**) patterns of PU composites under a N_2_ atmosphere.

Heat resistance is crucial for PU application. In this section, the impact of component B content on the thermal stability of PU samples was investigated. Figure 6 presents the TGA and DTG results for PU composites measured from 35 to 700 °C in a nitrogen atmosphere, with detailed data provided in Table 3. In Figure 6a, the T_5%_, T_30%_, and T_peak_ of the PU composites (S1–S5) with the addition of CSR were reduced compared to S0. This indicated that the addition of CSR particles leads to a decrease in the thermal stability of the PU composites. However, the thermal stability of the PU composites was slightly increased with the increasing of component B. The residues of S0, S1, S2, S3, S4, and S5 at 700 °C were 12.1%, 8.5%, 9.0%, 8.6%, 8.8%, and 9.8% of the initial mass, respectively. These implied that the incorporation of the CSR particles disrupted the polyurethane network. However, the increase in component B slightly improves the thermal stability of the PU composites [40]. In the DTG curves (Figure 6b), S0 shows a major peak at 328 °C and a minor peak at 475 °C, which may be related to the thermal decomposition of the soft and hard segments of the PU matrix [41,42]. When the CSR particles were introduced, not only the main peak but also some shoulder peaks appeared in the curves. This may be attributed to the thermal decomposition of the CSR particles. In contrast, the exothermic peaks after the addition of CSR were small, and the heat release rate was slow. It can be concluded that the addition of component B has a slowing effect on the exothermic rate of the polyurethane composites in an inert atmosphere and has an effect on the thermal stability of the material.

### 3.4. Thermo-Mechanical Behavior of the PU Composites

**Figure 7:**
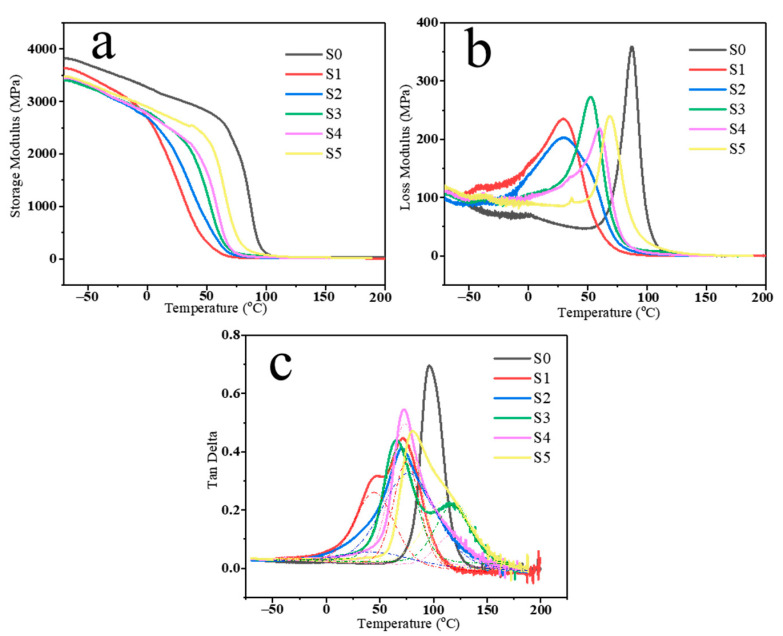
Temperature dependence of (**a**) storage modulus, (**b**) loss modulus, and (**c**) tan delta for PU composites.

To further clarify the influence of the network structure and CSR particles on dynamic stiffening, we analyzed the DMA data at 1 Hz for all PU composites, as illustrated in Figure 7. Typically, the storage modulus is associated with the material’s elasticity and serves as an indicator of its load-bearing capacity and stiffness. Generally, the storage modulus of polymers is influenced by temperature, fillers, molecular chain structure, and the interactions between chain segments [43,44]. As shown in Figure 7a, the storage modulus of the PU samples increased with the rising component B after the first glass transition temperature (Tg, which seems to exist in these PU composites). This may be due to the enhanced rigidity resulting from the increased presence of various polyols, CSR particles, and the restricted mobility of the network structure. All samples exhibited a continuous decrease as the temperature rose, primarily due to the softening of PU chains at higher temperatures. The loss modulus is typically associated with the filler and material viscosity, providing insight into the motion of polymer chains and the damping effects resulting from energy dissipation. Figure 7b illustrates the changes in loss modulus of the PU composites as a function of temperature. The loss modulus of the PU composites (S1–S5) decreased across the tested temperature range compared to S0, primarily due to the increased filler content and the formation of a cross-linked network [45]. The presence of CSR particles and the network structure hinders effective stress transmission, resulting in a reduction of the loss modulus. The loss factor, tan delta, represents the ratio between the loss modulus and the storage modulus, offering essential insights into the mechanical properties of the composite. The glass transition temperature (Tg) of the polymer is primarily associated with the delayed relaxation caused by the coordinated segmental motion of polymer chains. It is typically indicated by the temperature at which the peak occurs in the tan delta curve. Table 4 lists the Tg of different PU composites. As shown in Figure 7c, the main Tg of S0, S1, S2, S3, S4, and S5 are 96, 70, 72, 68, 72, and 80 °C, respectively. It is evident that the peak value for the PU composites decreased when compared to S0, indicating a reduction in the mobility of the PU chains in the presence of CSR particles. According to tan delta curves, it seems that some PU composites have two Tg peaks (S1–S5). Then, we fitted these curves with peak splitting. The relevant Tg data from the peak splitting fits are shown in Table 4. For S1 and S2 samples, low-content CSR particles and polyols lead to reduced cross-linking of the polyurethane network, and the first Tg appears at about 45 °C. When CSR is excessive, agglomeration occurs. It leads to a deterioration of the toughness, while the main Tg increases and a second Tg occurs. Therefore, the enhancement of Tg values with the rising content of component B can primarily be ascribed to the network structure and CSR particles, which restrict the movement of the PU chains.

### 3.5. Temperature and Weathering Resistance of PU Composites

**Figure 8:**
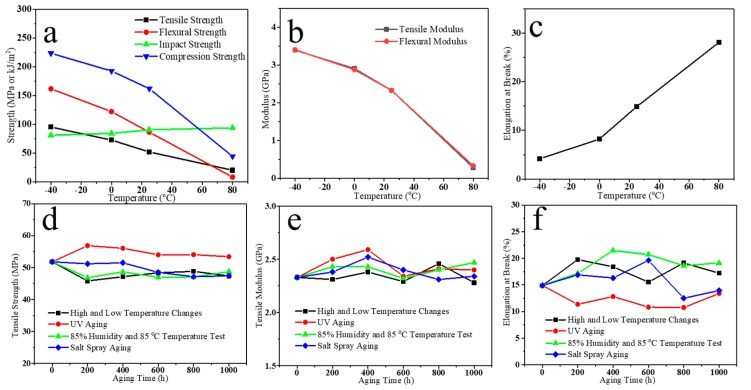
(**a**) Strength, (**b**) modulus, and (**c**) elongation at break changes of S3 samples at different temperatures; (**d**) tensile strength, (**e**) tensile modulus, and (**f**) elongation at break changes of S3 samples under different environmental conditions.

From these analyses above, we have identified the good mechanical properties and thermal stability of the composites represented by the S3 sample. In order to explore the potential use of PU composites in different areas, we further investigated mechanical properties of the S3 samples under different temperatures and environmental conditions. The test data and results are shown in Figure 8a–f and Table 5 and Table 6. In Figure 8a, the tensile, flexural, and compressive strengths of the S3 composites decreased with increasing temperature, which were 20.23, 28.08, and 44.31 MPa at 80 °C, respectively, while the impact strength of the S3 composites was the highest at 80 °C with 93.69 kJ/m^2^. Meanwhile, the tensile and flexural modules of the S3 samples decrease dramatically with increasing temperature in Figure 8b, while the elongation at break increases greatly in Figure 8c. This indicates that high temperature has a significant effect on the strength of the S3 composites. It may be attributed to the fact that high temperatures enhance the motility of the molecular chains and weaken the rigidity of the PU cross-linking network, leading to a decrease in tensile strength and modulus. At the same time, high temperatures increase plastic deformation and internal relaxation of the material, which strengthens the damping effect and energy absorption, resulting in improved impact strength and elongation at break. Overall, the S3 composites show good mechanical properties at different temperatures, ranging from −40 to 20 °C. Furthermore, we have examined the weathering resistance of the S3 samples through different tests. As shown in Figure 8d–f, all the mechanical properties of S3 samples change little after aging 1000 h. It shows that S3 samples can resist extreme testing environments, such as high and low temperature changes, UV aging, humidity and heat aging, and salt spray aging, which has potential and value for practical application.

### 3.6. Structural Adhesive Application of PU Composites

**Figure 9:**
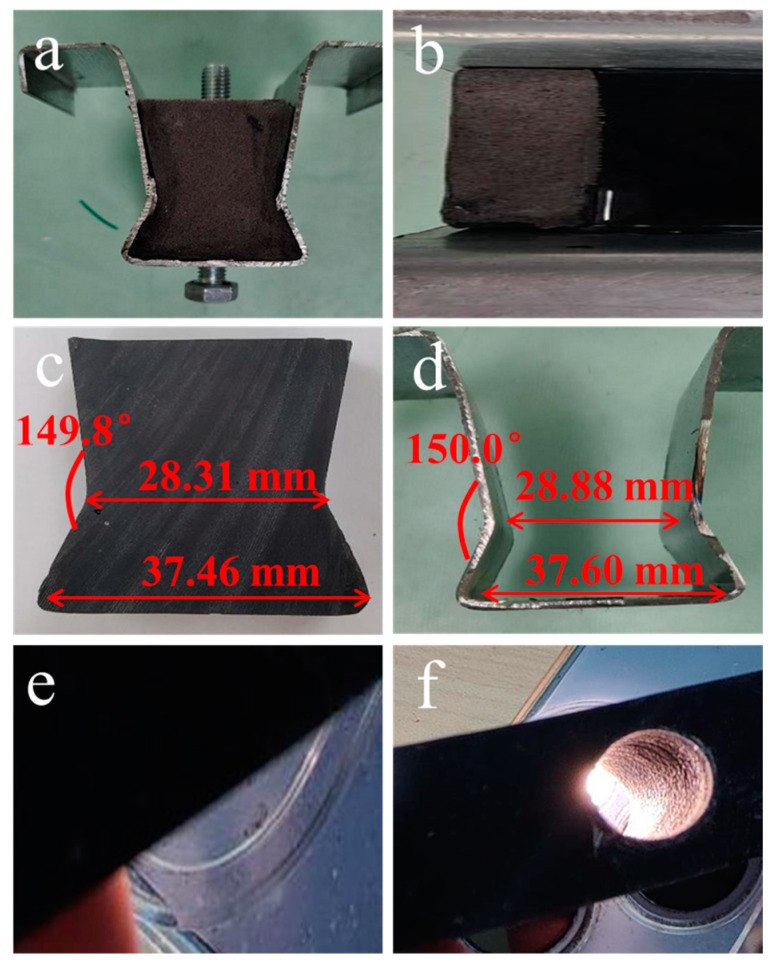
Digital photograph of: (**a**) structural adhesive application of PU composite; (**b**) structural adhesive bonded to a metal surface; dimensions of (**c**) PU structural adhesive block and (**d**) a metal frame; (**e**) surface and (**f**) inside of the PU structural adhesive block.

In order to verify the practical application potential of this PU composite, we poured the prepared S3 sample into a self-made metal frame. A digital photograph of the cross-section after complete curing is shown in Figure 9a. We can find that the interface between the PU structural adhesive and the metal frame was tightly bonded after complete curing, and there were no obvious air bubbles or detachment in Figure 9b. Carefully separating this structural adhesive block from the metal frame, it was found that the dimensional difference between these two was extremely minor, as shown in Figure 9c,d. This indicates that the PU structural adhesive is not only able to cure quickly and form a tight structure with the metal frame, but also able to vary with the shape and size of the metal frame with essentially no shrinkage. We further observed the surface and interior of the structural adhesive block, as shown in Figure 9e,f. Not only is the surface of the structural adhesive block homogeneous and dense, but the interior is smooth and no air holes have been created. We believe this PU structural adhesive with outstanding performance will be applied on a large scale in the future for repairing and bonding composite materials, wind turbine blade parts, and other plastic products.

## 4. Conclusions

In this work, PU composites with excellent mechanical properties were prepared successfully via regulating the cross-linking structure and the dispersion of CSR particles. Various types of polyols were selected to improve the cross-linking curing density of the PU and to enhance the intermolecular forces, which can achieve high strength and stability of the composites. Furthermore, the cured composite has ultra-high toughness and impact resistance due to the well-dispersed CSR rubber particles. The impact strength was increased from 52.0 kJ/m^2^ to 90.4 kJ/m^2^, and the elongation at break was increased from 6.1% to 14.9%. In addition, the composites have good thermal stability and weather resistance. They are able to maintain good mechanical properties under various temperatures and environmental conditions. Due to the addition of a catalyst, the curing speed of the composite material is extremely fast, less than 75 s. It can be used to connect structural parts in a short period of time and can also be used for the maintenance of building structures, renovation, and the surface closure of small cracks in reinforcement works. This composite structural adhesive is not only able to achieve high mechanical strength in a short period of time, but also has ultra-high toughness when fully cured. This study presents a new strategy to prepare polyurethane composites with excellent mechanical properties for structural adhesive applications.

## Figures and Tables

**Table 1:** Composition of components A and B (200 wt% total).

Component A	Component B				
PM200	DMN400	DDL1000	NX9008	NJ403	HP2210
	35.0	12.0	20.0	5.0	11.0
100	MOCA	T120	BYK-X	BR301	FD3110
	5.0	0.2	1.4	0.4	10.0

**Table 2:** Compositions of PU composites.

Samples	Component A	Component B + FD3110	Component A/B Ratio	FD3110 (wt%)
S0	100	80 (80.0 + 0.0)	100/72.0	0.0
S1	100	64 (57.6 + 6.4)	100/57.6	3.9
S2	100	72 (64.8 + 7.2)	100/64.8	4.2
S3	100	80 (72.0 + 8.0)	100/72.0	4.4
S4	100	88 (79.2 + 8.8)	100/79.2	4.7
S5	100	96 (86.4 + 9.6)	100/86.4	4.9

**Table 3:** TGA data of PU composites under a N_2_ atmosphere.

Samples	T_5%_ (°C)	T_30%_ (°C)	T_50%_ (°C)	T_peak_ (°C)	Residue at 700 °C (wt%)
S0	299	335	346	342	12.1
S1	262	331	361	328	8.5
S2	269	330	360	328	9.0
S3	269	330	358	328	8.6
S4	274	330	359	328	8.8
S5	278	333	367	329	9.8

**Table 4:** Tg values of PU composites according to DMA curves.

Samples	Tg1 (°C)	Tg2 (°C)
S0	96	-
S1	45	70
S2	46	72
S3	68	118
S4	72	118
S5	80	119

**Table 5:** Mechanical properties of the PU composites at different temperatures.

Temperature (°C)	Tensile Strength (MPa)	Flexural Strength (MPa)	Impact Strength (kJ/m^2^)	Compression Strength (MPa)	Elastic Modulus (GPa)	Elongation at Break (%)
−40 °C	95.16	161.51	80.90	223.40	3.41	4.14
0 °C	72.54	122.01	84.08	192.50	2.88	8.23
20 °C	51.82	86.3	90.40	161.91	2.33	14.9
80 °C	20.23	28.08	93.69	44.31	0.33	28.08

**Table 6:** Mechanical properties of the PU composites under different conditions.

	High and Low Temperature Change Test			UV Aging Test		
Aging Time (h)	Tensile Strength (MPa)	Elastic Modulus (GPa)	Elongation at Break (%)	Tensile Strength (MPa)	Elastic Modulus (GPa)	Elongation at Break (%)
0 h	51.82	2.33	14.91	51.82	2.33	14.91
200 h	45.83	2.31	19.78	56.88	2.5	11.36
400 h	47.16	2.38	18.41	56.07	2.59	12.83
600 h	48.39	2.29	15.54	54.01	2.34	10.84
800 h	48.85	2.46	19.15	54.07	2.41	10.76
1000 h	47.39	2.28	17.21	53.43	2.40	13.40
	85% Humidity and 85 °C Temperature Test			Salt Spray Aging Test		
0 h	51.82	2.33	14.91	51.82	2.33	14.91
200 h	46.85	2.43	17.18	51.21	2.38	16.90
400 h	48.68	2.43	21.47	51.52	2.52	16.32
600 h	46.97	2.32	20.72	48.53	2.40	19.61
800 h	47.06	2.40	18.62	47.16	2.31	12.51
1000 h	48.72	2.47	19.15	47.43	2.31	13.95

## Data Availability

The original contributions presented in the study are included in the article/Supplementary Materials, further inquiries can be directed to the corresponding author/s.

## Supplementary Materials

The following supporting information can be downloaded at: https://www.mdpi.com/article/10.3390/polym16233263/s1, Figure S1: The CSR particle size distribution and photographs of the dispersion of the CSR particles in component B before (a) and after (b) swelling; Figure S2: TEM image of the CSR particle; Figure S3: Comparisons of mechanical properties of different PU composites; Table S1: Mechanical and physical properties of the PU composites; Figure S4: Different structural adhesive applications: rectangular structural adhesive measuring: (a) 35 mm × 35 mm; (b) 50 mm × 50 mm; (c) 50 mm × 50 mm at different heights. (d1~d4) Digital photograph of the self-made metal frame with structural adhesive; Figure S5: Relevant chemical formula (a) and reaction equations (b) in this work.
