# Supplementary material for: Regulating Cross-Linking Structure and Dispersion of Core-Shell-Rubber Particles in Polyurethane Composite to Achieve Excellent Mechanical Properties for Structural Adhesive Application
Source: Polymers (Basel). 2024 Nov 23;16(23):3263. doi: 10.3390/polym16233263 (PMC11644690; doi:10.3390/polym16233263)
Supplement: Supplementary file 1 [file polymers-16-03263-s001.zip › polymers-3247738-supplementary.pdf]

Supporting Information

Regulating Cross-Linking Structure and Dispersion of Core-Shell-Rubber Particles in Polyurethane Composite to Achieve Excellent Mechanical Properties for Structural Adhesive Application

Zijin Jiang [1,2], Lingtong Li [1], Luoping Fu [2], Yingte Xu [2], Lin Zhang [2], Hong Wu [1,*] and Shaoyun Guo [1]

[1] The State Key Laboratory of Polymer Materials Engineering, Polymer Research Institute of Sichuan University, Chengdu 610065, China; jiangzijin@dongfang.com (Z.J.); lingtongli1994@163.com (L.L.); nic7702@scu.edu.cn (S.G.)
[2] Sichuan Dongshu New Materials Co., Ltd., Deyang 618000, China; fuluoping@outlook.com (L.F.); xu1998yt@outlook.com (Y.X.); dy_zlin@163.com (L.Z.)
* Correspondence: wh@scu.edu.cn

The swelling of PMMA shell in butanone resulted in an increase in the size of the CSR particles and exposure of more hydroxyl groups, which regulated the dispersion in component B. The particle size distribution of the CSR particles before and after swelling was characterized by a laser particle size shape analyzer, as shown in Figure S1. The particle size distribution of the dissolved CSR particles broadens. Photographs of the dispersion of the CSR particles in the component B before and after swelling are also in Figure S1. It can be found that the dissolved CSR particles have well dispersion in the component B, and there is no obvious agglomeration and deposition phenomenon.

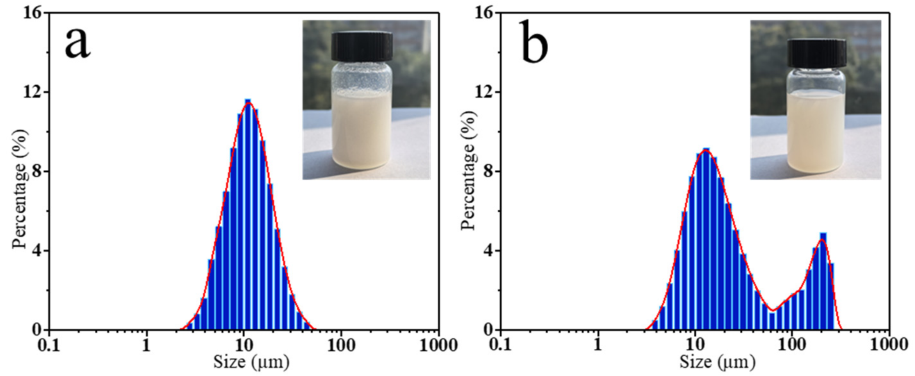

Figure S1. The CSR particles size distribution and photographs of the dispersion of the CSR particles in the component B before (a) and after (b) swelling.

The TEM image of the CSR particle is shown in Figure S2. The morphology of this particle can be found as microspheres with a diameter of about 10 μm. Due to the large particle size of the CSR particles, we could not further test its nuclear structure in the TEM test. However, from the information reflected by the provider, CSR is actually a CSR particle composed of a PMMA shell and a PBR core.

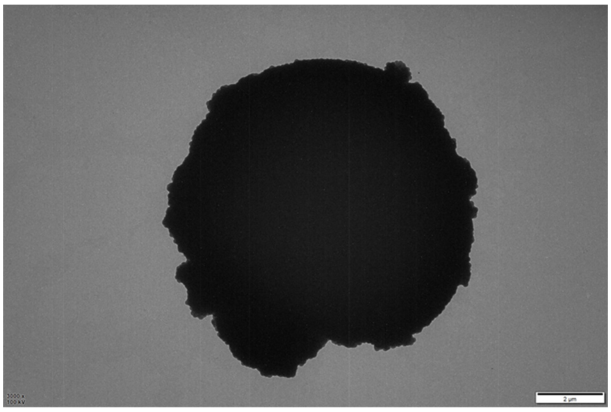

Figure S2. TEM image if the CSR particle

In Figure S3, we compare the mechanical properties of the fully cured S0 sample, S3 sample and the S3-1 sample (cured for 30 minutes). Compared to the S3 sample, the short curing time of the S3-1 sample resulted in an unstable cross-linking structure of the PU structural adhesive, which led to a different decrease in tensile strength, impact strength and tensile modulus. But its elongation at break is greatly improved. Compared to the S0 sample, the tensile strength and tensile modulus of the S3-1 sample are partially reduced, but the impact strength and elongation at break are improved. This indicates that even after a short curing time, the S3 samples still have excellent mechanical properties. More than 70% of the structural strength can be achieved after curing for 30 min at room temperature. The prepared PU structural adhesive can meet the requirements of structural bonding transit and improve the production efficiency. This is very important for the practical application of structural adhesives.

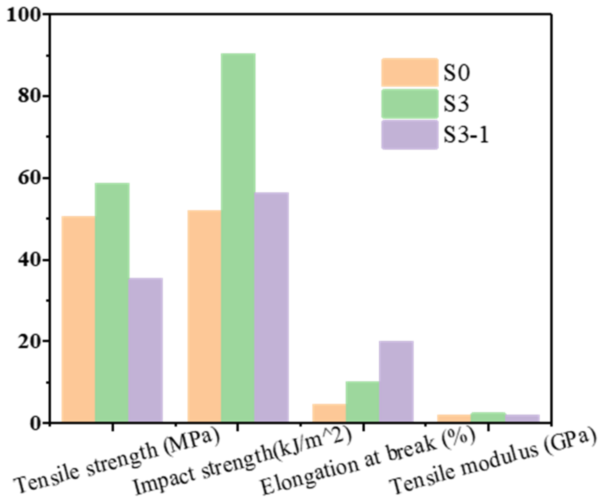

Figure S3. Comparisons of mechanical properties of different PU composites.

In order to verify the mechanical performance of PU composites, we investigated the mechanical properties of the pouring samples at room temperature. The test results are shown in Figure 3a–f, and the data is listed in Table S1. The various mechanical properties of the PU samples showed different trends of increase and decrease with increasing B-component content.

Table S1. Mechanical and physical properties of the PU composites.

Samples	Tensile Strength (MPa)	Impact Strength (kJ/m^2)	Flexural Strength (MPa)	Elongation at Break (%)	Elastic Modulus (GPa)	Flexural Modulus (GPa)

S0	50.6 ± 2.2	52.0 ± 6.7	62.1 ± 6.5	6.4 ± 0.7	2.5 ± 0.1	2.0 ± 0.1
S1	42.3 ± 2.0	80.3 ±11.4	36.1 ± 3.8	4.7 ± 0.8	2.0 ± 0.1	1.2 ± 0.1
S2	44.2 ± 2.1	85.8 ± 8.5	59.4 ± 6.8	7.9 ± 0.7	2.1 ±0.1	1.9 ± 0.1
S3	51.8 ± 2.5	90.4 ± 9.6	86.3 ± 2.0	14.9 ± 1.0	2.3 ± 0.1	2.4 ± 0.1
S4	46.9 ± 2.1	76.3 ± 11.4	93.9 ± 3.2	7.4 ± 0.6	2.1 ± 0.1	2.6 ± 0.1
S5	57.8 ±2.5	81.1 ± 11.8	95.0 ± 1.5	4.1 ± 0.7	2.5 ± 0.1	2.5 ± 0.1

Figure S4 shows the different structural adhesive applications in this work. Repeated experiments on the structural adhesive with different metal frames nave been done.

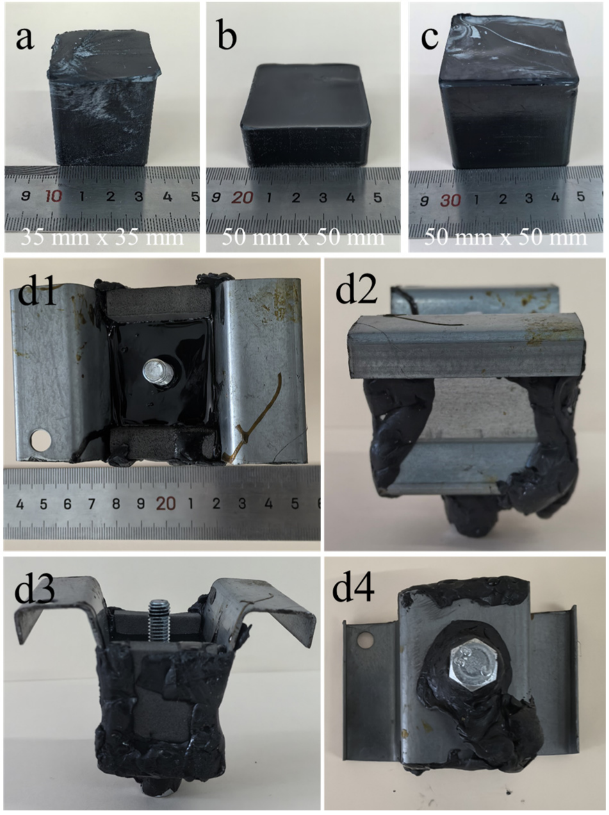

Figure S4. Different structural adhesive applications: rectangular structural adhesive measuring: (a) 35 mm x 35 mm; (b) 50 mm x 50 mm; (c) 50 mm x 50 mm at different heights. (d1~d4) Digital photograph of the self-made metal frame with structural adhesive.

The structural formulae of some of the commercially confidential polyols are speculative and may not be entirely correct. Relevant chemical formula and reaction equations in this work are shown Figure S5. The reactions of various types of polyols with MDI (PM200) can be classified into two groups, as shown in Figure S5b. Eventually, the reaction is complete and the PU is cured, forming a crosslinked structure.

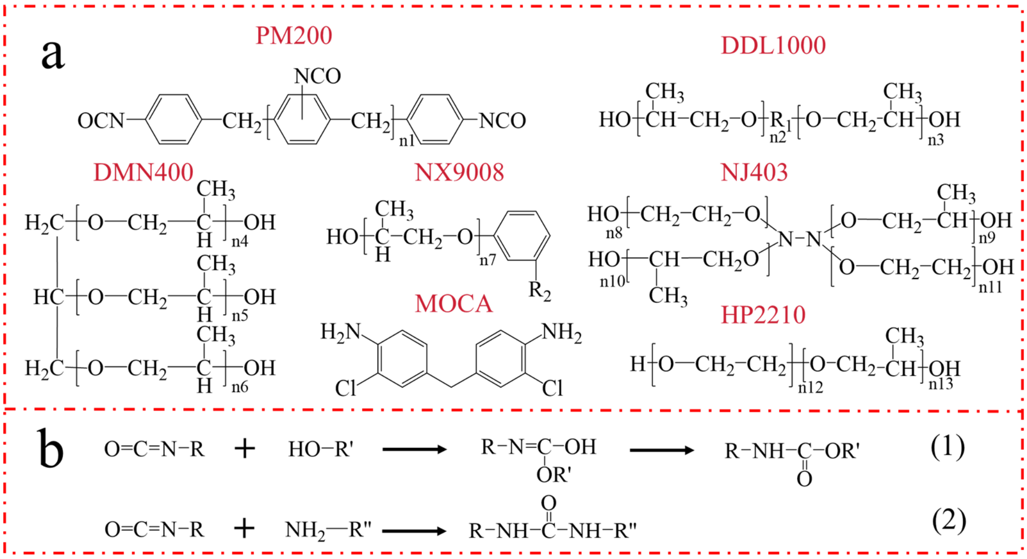

Figure S5. Relevant chemical formula (a) and reaction equations (b) in this work.
